# Paraurethral Leiomyoma in a Postmenopausal Woman: First European Case

**DOI:** 10.1155/2015/542963

**Published:** 2015-03-24

**Authors:** Susy Shim, Camilla Skovvang Borg, Huda Galib Majeed, Peter Humaidan

**Affiliations:** ^1^Department of Gynecology and Obstetrics, Aalborg University Hospital, Reberbansgade 15, 9000 Aalborg, Denmark; ^2^Department of Gynecology and Obstetrics, Viborg Regional Hospital, Heibergs Allé 4, 8800 Viborg, Denmark; ^3^The Fertility Clinic, Skive Regional Hospital, Resenvej 25, 7800 Skive, Denmark; ^4^Faculty of Health, Aarhus University, Nordre Ringgade 1, 8000 Aarhus, Denmark

## Abstract

Leiomyomas are benign tumors extending from smooth muscle cells and only few cases of paraurethral leiomyomas have been described in the literature. They are often seen in the reproductive age and around 50% of the cases are asymptomatic. We describe a 59-year-old woman with a solid mobile tumor below the symphysis revealed at a gynecological examination. Transvaginal ultrasound and MRI confirmed the tumor and excision of the paraurethral tumor was carried out. The histological examination showed a benign paraurethral leiomyoma. The postoperative period was characterized by urethral pain as well as vaginal leakage of urine.

## 1. Introduction

Leiomyomas are benign tumors extending from smooth muscle cells. They are the most common type of tumors in the female reproductive organs, but they are rare outside the uterus. Only few cases of paraurethral leiomyomas (PL) have been described in the literature. They are often seen in the reproductive age and around 50% of the cases are asymptomatic.

We hereby present the first European case of a paraurethral leiomyoma in a postmenopausal woman.

## 2. Case

A 59-year-old woman was referred to a gynecological outpatient clinic suspected to have an ovarian cyst. Some months earlier she had been examined at the general surgery department due to abdominal pain.

The patient had previously undergone a hysterectomy and was medically treated for diabetes. She was diagnosed with diverticulosis and the suspicion of a cyst in the left ovary. She had experienced constipation problems and pain in the lower left side of the abdomen. The pain increased during movement and when eating. The constipation was improved by lactulose treatment, and as a result she only suffered from abdominal pain during physical activity. Importantly, she also reported occasional incontinence, alternating with difficulties emptying the bladder.

A gynecological examination revealed a solid mobile tumor below the symphysis, measuring 3 × 3 cm. A transvaginal ultrasound examination showed two normal ovaries and normal findings in the lower pelvis. In contrast, a tumor measuring 2.8 × 2.3 cm closely related to and intruding into the urethral wall was seen.

**Figure 1:**
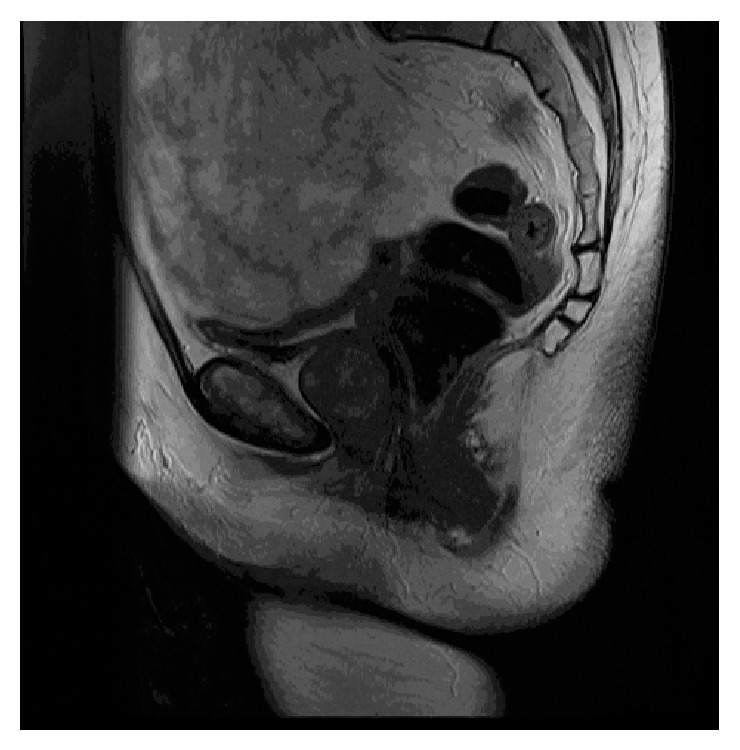
MRI of paraurethral leiomyoma in relation to urethra.

A magnetic resonance imaging (MRI) revealed a sharply defined tumor, measuring 3 × 3 cm closely related to the vaginal vault (Figure 1). The tumor caused an impression of the urethra, but invasive growth was not suspected. A transvaginal biopsy of the process showed smooth muscle tissue without signs of malignancy.

Subsequently, an excision of the paraurethral tumor was carried out in collaboration between urologists and gynecologists. In the beginning of the operation a cystoscopy was performed. A little diverticel was seen in the urethra but there was no observed connection between the paraurethral tumor and the urethra. The operation was performed with only local anesthesia. Lidocaine with adrenalin was injected into submucosa in the anterior wall of vagina. The mucosa was divided lengthwise and the tumor was dissected free. During the dissection a minor lesion of the bladder occurred and lesion was sutured in two layers. Injection with methylene blue fluid confirmed no leakage in the lesion. Finally the pubocervical fascia and the mucosa in vagina were sutured. The patient was catheterized for one week and got antibiotic treatment both preoperatively and postoperatively for one week.

The postoperative period was characterized by urethral pain as well as vaginal leakage of urine. A subsequent gynecological examination revealed two vesicovaginal fistulas, which were sutured. Following this the leakage stopped and the patient was free of symptoms.

**Figure 2:**
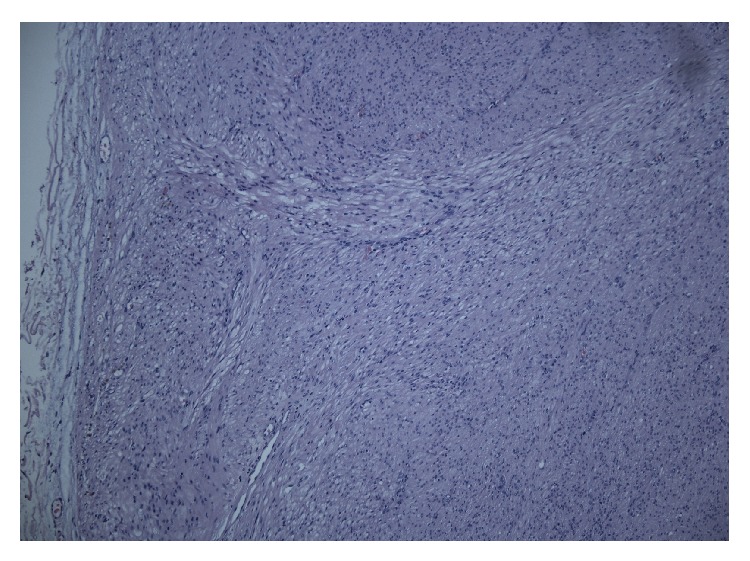
Rem of leiomyoma in relation to urethra, HE-stain.

The histological examination showed a benign leiomyoma (Figure 2).

## 3. Discussion

PL is rare benign tumors originating from smooth muscle cells [1, 2]. Leiomyomas occur throughout the genitourinary system, most commonly in the renal capsule [1]. They can occur in both sexes but are predominantly found in women and primarily during the reproductive age [3]. In total less than 30 cases of PL have been documented in Europe since 1977. PL cases are often asymptomatic, but larger tumors might cause symptoms such as pain, voiding problems, and dyspareunia [3]. One case presented with profuse vaginal bleeding [4].

Despite the fact that PLs are benign tumors, most studies recommend that the PL is removed in order to rule out a leiomyosarcoma [5]. Other differential diagnosis should be made with urethral diverticula, vaginal cysts, urethrocele, Gartner's cysts, and Skene's duct abscess [6].

Without an operation the patient might still experience pressure symptoms with risk of deterioration.

Although a MRI can give an idea of whether a leiomyoma or a leiomyosarcoma should be suspected, the final diagnosis is histological.

Leiomyomas typically appear as round and well-defined structures. There is a low T1 signal intensity and a low to intermediate T2 signal intensity [3]. Degenerated areas, though, may show high intensity. In contrast, leiomyosarcomas are irregular and heterogeneous in structure and with areas of necrosis or hemorrhage [3, 7]. Furthermore, they have a high level of T2 signal intensity. Findings from the MRI-scan may be compared to findings from the transvaginal biopsy, allowing the surgeon to optimize the operative procedure.

It is believed that ovarian hormones stimulate the development of leiomyomas because they mainly occur in the reproductive age [2, 8]. It is supported by reports of enlargement during pregnancy and shrinkage after delivery, and it has also been demonstrated in one case with presence of estrogen and progesterone receptors with immunohistological chemical examination [1, 7]. But some authors have suggested that ovarian hormones do not influence the development because, as in our case, the tumor also occurs in a postmenopausal woman. So further studies with larger population are needed to make any conclusions.

In our case neither any hormonal studies nor immunohistological chemical examination was performed.

In conclusion, we present the first PL case reported in a postmenopausal European woman. Our case demonstrates the importance of a thorough vaginal exploration, including the entire vagina when uncharacteristic pain occurs in the lower pelvis in combination with voiding problems. even in postmenopausal women.
